# The Mediating Effect of Self-Control on Depression and Tendencies of Eating Disorders in Adolescents

**DOI:** 10.3389/fpsyt.2021.690245

**Published:** 2021-12-17

**Authors:** Hong-Juan Li, Jie Li, Meng Qi, Tian-He Song, Jing-Xu Chen

**Affiliations:** ^1^Beijing Hui-Long-Guan Hospital, Peking University Hui-Long-Guan Clinical Medical School, Beijing, China; ^2^Rizhao People's Hospital of Shandong Province, Rizhao, China; ^3^Department of Psychology, Chengde Medical University, Chengde, China

**Keywords:** self-control, depression, eating disorder, mediating effect, adolescents

## Abstract

Self-control is very important for the adaptation among adolescents. It is associated with depression and tendencies of eating disorders. This study aimed to investigate the relationship between the two and the mediating role of self-control for adolescents. In total, 1,231 adolescents (11–18 years) participated in this study. Self-control, depression, and tendencies of eating disorders were evaluated using the Dual-Mode of Self-Control Scale (DMSC-S), 11-item Kutcher Adolescent Depression Scale (KADS-11), and Eating Attitudes Test (EAT-26). The correlations among these factors were analyzed using mediating effect models. Girls had higher scores on the both subscales (impulse system and control system) of DMSC-S (*P* < 0.001). Those between 15–18 years had higher scores on impulse system than those between 11–14 years (*P* < 0.001). A significant mediating effect (12.8%) of the impulse system was observed between depression and tendencies of eating disorders in adolescents.

## Introduction

Depression is the leading cause of illness and disability among adolescents (1). Adolescent depressive disorder, which is a group of mental illnesses mainly characterized by negative feelings, may be accompanied by varying degrees of cognitive and behavioral changes, psychotic symptoms, impulsive non-suicidal self-injury (NSSI), and impulsive suicide, among others (2–5). The 2014 Ontario Child Health Study reported that the 6 month prevalence of possible major depressive disorder (MDD) was 7.5% for adolescents (12–17 years old) (6). The overall prevalence of depressive symptoms among Chinese adolescents was 14.81% (7). Based on these findings, adolescent depressive disorder can considered to be a common social problem and should be paid more attention.

Eating disorder (ED) symptoms are highly prevalent in adolescents and are regarded as one of the most important ED precursors clinically (8). An ED, which includes anorexia nervosa (AN), bulimia nervosa (BN), and binge eating disorder (BED) (9), is characterized by impulsive eating or following diets compulsively, and is the result of the interaction between specific cultural and psychosocial factors. In adolescents, the lifetime prevalence of AN, BN, and BED was 0.3, 0.9, and 1.6%, respectively (10).

Depression comorbid with ED is common and can increase both conditions' severity and chronicity (11). Studies have shown 80% of patients with ED have emotional disorders (12), with depression being the most common (12). Compared with ED patients without other mental disorders or with anxiety, the symptoms of ED patients with depression are more complex (13). There have been some studies on the relationship between depression and ED; however, the mechanism of the comorbidity remains unknown.

Depression may put the patient at a risk of developing a chronic ED (14, 15). MDD frequently co-occurs with BED or BN, and there is evidence that depression predicts the onset of ED and loss of controlled eating in adolescent girls (16). But how do comorbidities affect patients remain unknown. Impulsivity has been found to be a significant contributing factor for depression and ED (17), and has been long associated with the former (18)—patients with MDD show poor impulse control (19). A study of mood disorders and impulsivity reported that people with depression had significantly higher impulsivity scores than healthy individuals (20). Several studies have shown that people with BED are characterized by increased impulsivity (21, 22), and it has been associated with an increased risk of ED development (23). Individuals with ED have many impulsive behaviors, including substance abuse (24), NSSI (25), and shoplifting (26).

The Dual Modes of Self Control scale (DMSC-S) includes impulse and control systems (27). Hofmann et al. (28) first proposed DMSC-S, believing that DMSC-S includes: ① impulse system, which is the cause of impulsive behavior. When faced with temptation, it will automatically arouse a corresponding impulsive behavior, supporting individuals to choose instant gratification; ② The control system is the cause of higher order psychological activities in the face of temptation, including thoughtful evaluation and inhibition criteria, encouraging individuals to choose to wait to achieve the predetermined goal. The individual's final choice depend on which system plays a greater role in achieving a predetermined goal. The level of self-control was assessed in this study. Strong impulses or weak controls can lead to impulsivity (28), and weak controls may lead to antisocial outcomes during youth (29). Recent evidence has indicated that the control and impulse systems are different and there is only a moderate negative correlation between them (30). Several studies have shown that the dual-systems model fits the data significantly better than a one-dimension alone (30, 31).

While few studies have explored the relationship between depression and ED, the effects of depression on ED through self-control systems have not been studied. Therefore, the relationship among depression, ED, and self-control needs to be further studied. This study has two hypotheses— first, depression is a predictor of ED; and second, depression can directly predict the severity of ED and also indirectly affect ED through self-control.

## Materials and Methods

### Participants

This cross-sectional study was conducted between February 15 and May 15 2017, in three middle schools in Rizhao, Shandong Province, China. To obtain a representative sample, we randomly selected four schools. From each school, ninth grade classes were randomly chosen, and all students in the selected classes were asked to participate. These students' parents also agreed to participate in the study and signed informed consents. A total of 1,300 students participated in this survey, and 1,231 (94.7%) completed the questionnaire. Participation was voluntary, and participants were asked to do so anonymously. This study was approved by the Institutional Review Board of Beijing Huilongguan Hospital.

### Procedure

Our research team completed the assessment during school hours, and it lasted about 45 min. Questionnaires were collected on the spot. We designed a general questionnaire to investigate the age, grade, height, ideal weight, actual weight, and other general information of the participants and used the above information to calculate the ideal body mass index (BMI) and the actual BMI. BMI was calculated as weight in kilograms divided by height in meters squared.

The 11-item Kutcher Adolescent Depression Scale (KADS-11), a self-reported instrument, was initially applied to a Canadian population (32) to investigate depression. The language of this scale is easy to understand and it can quickly identify patients with depression (32). The items of the KADS-11 are constructed according to the frequency of depressive symptoms and core symptoms of depression. The severity levels are 0 (almost none), 1 (most of the time), 2 (most of the time), and 3 (all the time). The total score is the sum of the scores for each item, ranging from 0 to 33, and a score of ≥9 indicates that the respondent has depressive symptoms (validity and reliability of the Chinese version of the Kutcher Adolescent Depression Scale). The Cronbach's coefficient of the KADS-11 was 0.84 (32).

The DMSC-S was used to investigate a participant's self-control levels (28). It has 21 items, and each answer is measured on a five-point scale, from 1 = “Not at all true” to 5 = “Very true.” The DMSC-S includes the impulse system (12 items) and control system subscales (nine items). They contain three (impulsivity, easy distraction, and delay gratification) and two factors (problem-solving and future time view), respectively. The total score of each dimension was calculated, and the higher the score, the higher was the level of individual impulse system/control system. The Cronbach's coefficient of the scale was 0.82.

The Eating Attitudes Test (EAT-26) was used to assess cognitive, emotional, and behavioral predispositions in eating (33). The EAT-26 is a 26-item self-report instrument, with each item having a six-point scale, based on the severity of symptoms: from 1 (“never”) to 6 (“always”). The total score is the sum of the scores for each item. A total score ≥20 indicates abnormal eating behavior (34), and the higher the score, the higher are the risk of tendencies of ED (35, 36). Kang et al. confirmed the reliability of rechecking and the factorial validity of the Chinese version of the EAT-26 (36). The Cronbach's coefficient of the EAT-26 was 0.80 (37).

### Data Analysis

Data were analyzed using SPSS version 24.0. Descriptive statistics were used to calculate the frequency of sample characteristics of the study population, and the results were presented as mean, standard deviation (SD), or percentage (%). The differences in socio-demographic characteristics, clinical characteristics, and self-control among the different groups were analyzed. For the data of normal distribution and non-normal distribution, the chi-square test was used for the classified variables, and the *T*-test and Mann-Whitney U test were used for continuous variables. The correlation among the each factor of DMSC-S, depression, and attitudes of disordered eating was evaluated using Spearman's correlation coefficient. Significance was based Bonferroni correction for 6 models *P* < 0.05/6 = 0.0083. Finally, we used the PROCESS v3.4 (by Andrew F. Hayes) macro (38) for SPSS to perform the mediation analysis. Random sampling was set 5,000 times. Under the 95% confidence interval, the sampling method selected the non-parametric percentile method of deviation correction. A *P*-value of < 0.05 indicated a significant difference.

## Results

The average age of the 1,231 participants was 14.53 ± 1.38 years old, and 679 (55.2%) were aged 11–14 years, while 552 (44.8%) were aged 15–18 years. The sample consisted of 587 (47.6%) males and 644 (52.3%) females. Of these, 523 (42.5%) met the criteria for depression and 106 (8.6%) were at a risk of developing ED. In all, 62 (11.9%) patients with depression met the criteria for ED. Relevant socio-demographic and clinical data of the participants are summarized in Table 1. This study found that girls had higher scores on the both subscales of DMSC-S than boys. Among all participants, the 15–18 years group had higher scores on the impulse system than the 11–14 years group. Participants residing in urban areas also had higher scores on the both subscales than those in rural areas. Compared with participants who had a normal perceived body weight, participants who were perceived to be underweight or overweight had higher scores on the both subscales. Participants with depressive symptoms had higher scores on the both subscales than participants who did not have depressive symptoms. Participants who met the ED criteria had higher scores on the impulse system than those who did not meet the ED criteria (see Table 2). In the correlation analysis, the each factor of control system (future time perspective and problem solving) were positively correlated with sex in participants with depressive symptoms. Among participants with tendencies of ED, sex was positively correlated with each factor of impulse system (impulsivity, distractibility and poor delay of gratification) (see Table 3).

**Table 1:** Socio-demographic characteristics and association with depression and risk of disordered eating attitudes (*N* = 1,231).

**Variables**	***n***	**%**	**Depression**			**Disordered eating attitudes**		
			***n***	**%**	***P***	***n***	**%**	***P***
Gender					0.872			**<0.001**
Male	587	47.6	248	42.2		32	5.5	
Female	644	52.3	275	42.7		74	11.5	
Age group					0.380			0.073
11–14 years	679	55.2	290	42.7		48	7.1	
15–18 years	552	44.8	228	41.3		56	10.1	
Region					0.089			**<0.001**
Rural	670	54.4	299	44.6		40	6.0	
Urban	561	45.6	223	39.8		66	11.8	
Body weight perception					0.908			**<0.001**
Normal weight	192	15.6	165	85.9		23	12.0	
Underweight	334	27.1	138	41.3		7	2.1	
Overweight	705	57.3	220	31.2		76	10.8	
Depression	523	42.5	–	–	–	62	11.9	**<0.001**
Disordered eating attitudes	106	8.6	62	11.9	**<0.001**	–	–	–

*P < 0.001 means the difference is statistically significant*.

**Table 2:** Relationship between socio-demographic and clinical characteristics of DMSC-S (*N* = 1,231).

**Variables**	**Impulse system**		**Control system**	
	**M, SD**	***P***	**Median (IQR)**	***P***
Gender		**<0.001**		**<0.001**
Male	29.76, 8.78		29 (25, 33)	
Female	30.98, 8.30		30 (26, 33)	
Age group		**<0.001**		**0.001**
11–14 years	29.36, 8.24		29 (25, 33)	
15–18 years	31.63, 8.73		29 (25, 32)	
Region		**<0.001**		**<0.001**
Rural	30.54, 8.53		29 (25, 33)	
Urban	30.27, 8.57		29 (26, 33)	
Body weight perception		0.158		**<0.001**
Normal weight	30 (24, 36)		30 (26, 33)	
Underweight	29 (25, 35)	**<0.001**	28 (25, 32)	**<0.001**
Overweight	31 (25, 37)	**<0.001**	29 (25, 33)	**<0.001**
Depression		**<0.001**		**<0.001**
Yes	31.03, 9.03		30 (26, 33)	
No	29.95, 8.13		29 (25, 32)	
Disordered eating attitudes		**<0.001**		**<0.001**
Yes	36.55, 8.38		28 (24, 33)	
No	29.69, 8.27		29 (26, 33)	

*P < 0.001 means the difference is statistically significant*.

**Table 3:** Correlation factor analysis of the each factor of DMSC-S.

**Variables**	**Depression**		**Disordered eating attitudes**	
	**Male**	**Female**	**Male**	**Female**
Future time perspective	0.121^**^	0.161^**^	−0.029	−0.109^**^
Problem solving	0.134^**^	0.165^**^	−0.103	0.049
Impulsivity	0.116^**^	0.058	0.290^**^	0.304^**^
Distractibility	0.036	0.027	0.206^**^	0.237^**^
Poor delay of gratification	0.080	0.012	0.199^**^	0.235^**^

** *P < 0.0083*.

**Figure 1:**
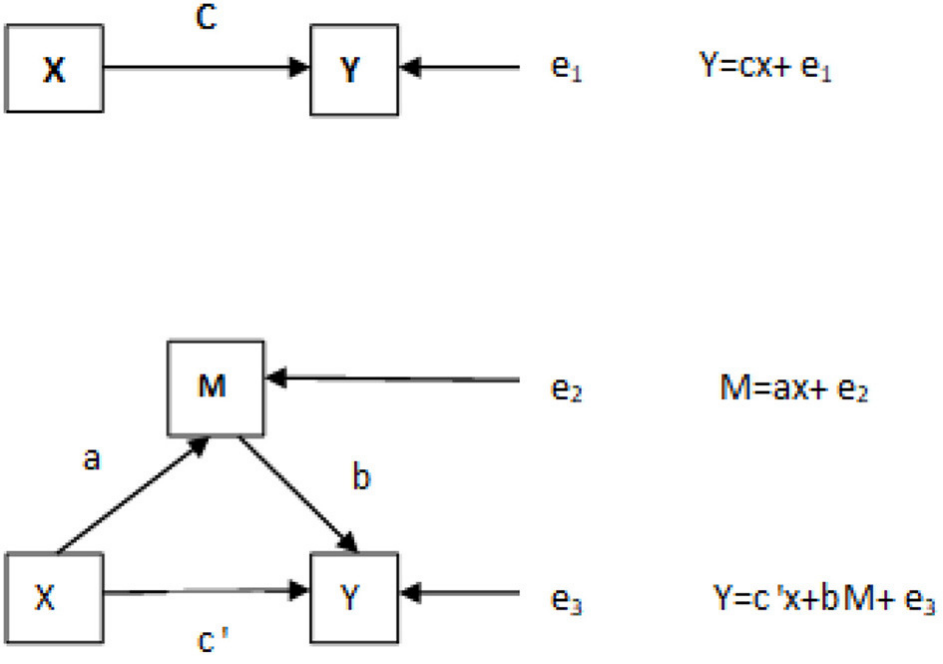
The mediating effect of DMSC-S impulse system on depression and disordered eating attitudes. X, Depression; M, DMSC-S impulse system; Y, disordered eating attitudes; a, the regression coefficient of X to M; b, the regression coefficient of M to Y; c, the regression coefficient of X to Y; e1, e2, e3, the regression residual.

With X as the score of KADS-11, M as the score of the DMSC-S impulse system, and Y as the score of EAT-26, the mediating effect measured by Process Model 4 was *Y* = −2.7937+ 0.37x +0.2911M. The equation was statistically significant (*P* < 0.01); partial regression coefficient B was 0.2911 and was significant (*P* < 0.01) with 95% CI (0.2435, 0.3387); partial regression coefficient C was 0.37 and significant (*P* < 0.01), with 95% CI (0.2738, 0.4665). The total effect was 0.4246, direct effect was 0.3701, and indirect effect was 0.0544. The intermediate effect accounted for 12.8%, bootstrap 95%CI (0.0116, 0.0999). The 95% confidence interval did not include 0, indicating that DMSC-S impulse system could predict ED directly and indirectly. The direct effect (0.3701) accounted for 87.2% of the total effect (0.4246), and the intermediate effect (0.0544) accounted for 12.8% of the total effect (0.4246) (Table 4). After controlling for age and sex, the median effect was 11.7%. The mediating effects of the DMSC-S impulse system on depression and ED are shown in Figure 1.

**Table 4:** Analysis of total effect, direct effect, and indirect effect.

	**Effect**	**Boot**	**Boot CI**	**Boot CI**	**Relative**
	**value**	**SE**	**lower**	**upper**	**effect value**
Total effect	0.4246	0.0517	0.3232	0.5259	
Direct effect	0.3701	0.0491	0.2738	0.4665	87.2%
Indirect effect	0.0544	0.0224	0.0116	0.0999	12.8%

## Discussion

Our study found a high incidence of depression and ED among adolescents. Some studies showed that one in ten adolescents had suffered from depression during the previous 12 months, and 14–18% were estimated to suffer from it throughout their lifetimes (39, 40). A study on the prevalence of mental disorders in China showed that the weighted lifetime prevalence of depressive disorders was 6.8% (41). Our study found that 42.5% of the participates met the criteria for depression. This result was much higher than the 14.81%, which we mentioned at the beginning of this article. The possible reason is that there is great pressure on students to excel academically during their high school and college entrance examinations. Consistent with previous studies (42, 43), our study also indicated that depression was highly prevalent in adolescents with ED. Depression and ED seem to have a circular relationship, in which they reinforce one another over time (44). This may be related to the levels of 5-HT in our bodies. Recent studies have shown that 5-HT receptor-binding alterations may lead to depression and ED (45, 46).

This study found that depression was not correlated with sex, age, region, or body weight perception among adolescents. This conclusion differs from previous studies. Previous studies (10, 28, 47) had found that boys were more likely to be depressed before mid-puberty, while the prevalence of depression is doubled in girls between 15 and 19 years. The possible reason is that our sample size is relatively small, and the subjects in this study came from a specific region of China, which might have affected the sample's representativeness. Further research with larger sample is needed to test if depression is correlated with sex. Tendencies of ED were correlated with sex, region, and body weight perception, but not with age. Previous studies have shown that the prevalence of ED in female adolescents is higher than that in males (48, 49). Young Chinese people generally believe that the thinner they are, the better. Thinness and prevention of weight gain are thus widely promoted by the Chinese media, especially for young women (50), and they may adopt various methods to control their weight, including fitness, diet, purging, etc., to cater to societal expectations. Therefore, they are more likely to suffer from ED.

Depression was positively correlated with each factor of control system. This means the higher the level of individual control system, the higher the risk of depression. There were no relevant reports on relationship between depression and control system. The possible reason is that adolescents are more rebellious and have ambivalence toward many things. The higher the adolescents' control over their emotions and behaviors, the more obvious the inner rebellion. If they can't find a proper outlet, they will feel more and more depressed. Some studies had reported no association between impulsivity and depression (51, 52), while others seem to support an association (53). The result of this study was consistent with the former. The tendencies of ED were positively correlated with all factors of the impulse system. This means the higher the level of impulsivity, the higher the risk of ED. This is consistent with previous study (54). It showed that the higher the impulsivity, the greater the risk of developing ED. Another study (55) found that the impulsivity was associated with binge eating. It was found, by mediating effects, that the impulse system could directly predict tendencies of ED. The direct effect (0.3701) and intermediate effect (0.0544) accounted for 87.2 and 12.8% of the total effect (0.4246), respectively (Table 4). After controlling for age and sex, the median effect was 11.7%. The mediating effect of the impulse system on depression and tendencies of ED is shown in Figure 1. Previous studies (54) have demonstrated that depression can cause tendencies of ED. Spence and Courbasson's research showed that participants could take action and use food as a coping mechanism to alleviate negative emotions (56). Konttinen's study indicated that depression is associated with mood and eating (57). However, there is no relevant report (domestically or internationally) to indicate whether it is directly or indirectly related through intermediary factors. In this study, we found that impulse systems have a mediating effect between depression and tendencies of ED.

Our findings have clinical implications by stressing the potential role of self-control in the development of ED, and hence as potential preventative therapeutic targets. Furthermore, findings point to the importance of therapeutic interventions targeting emotional regulation across these disorders, for example, interventions aimed at learning healthier strategies for coping with distress. Previous studies (58) had shown that cognitive behavioral therapy (CBT) could improve impulsivity and thus reducing the incidence of ED. The essential interventions are food-related cue exposure with response prevention and the development of self-control strategies. We can also provide CBT interventions for adolescents who are screened to be prone to ED to prevent the occurrence of ED. The present study had some limitations. First, the subjects in this study came from a specific region of China, which might have affected the sample's representativeness. Second, this study used a self-measuring scale. Finally, we choosed cross-sectional study. We did not find a causal relationship among depression, self-control and tendencies of ED. Prospective cohort study should be considered in the future.

## Conclusions

This study demonstrated that the impulse system might exert mediating effects between depression and tendencies of ED in adolescents. This indicates that guiding adolescents to control the degree of impulsivity and depression may be of great significance for preventing ED.

## Data Availability Statement

The raw data supporting the conclusions of this article will be made available by the authors, without undue reservation.

## Author Contributions

H-JL and JL: conceptualization and investigation. JL, MQ, and T-HS: methodology. MQ and T-HS: validation. H-JL: formal analysis and writing—original draft preparation. H-JL, MQ, and T-HS: data curation. JL and J-XC: writing—review and editing. J-XC, MQ, and T-HS: supervision. J-XC: project administration and funding acquisition. All authors contributed to the article and approved the submitted version.

## Funding

This research was funded by Capital Foundation of Medicine Research and Development (2018-3-2132) and Beijing Hospitals Authority Clinical Medicine Development of special funding support (XMLX202150).

## Conflict of Interest

The authors declare that the research was conducted in the absence of any commercial or financial relationships that could be construed as a potential conflict of interest.

## Publisher's Note

All claims expressed in this article are solely those of the authors and do not necessarily represent those of their affiliated organizations, or those of the publisher, the editors and the reviewers. Any product that may be evaluated in this article, or claim that may be made by its manufacturer, is not guaranteed or endorsed by the publisher.
